# Happy birthday: we are one year old

**DOI:** 10.1186/2046-4053-2-61

**Published:** 2013-08-06

**Authors:** David Moher, Lesley Stewart, Paul Shekelle

**Affiliations:** 1 Ottawa Hospital Research Institute, Centre for Practice-Changing Research (CPCR), The Ottawa Hospital, General Campus, 501 Smyth Rd, Room L1288, Ottawa, ON K1H 8L6, Canada; 2 University of Ottawa, Tabaret Hall, 75 Laurier Avenue East, Ottawa, ON K1N 6N5, Canada; 3 Department of Epidemiology and Community Medicine, Clinical Epidemiology Program, Ottawa Hospital Research Institute, University of Ottawa, Ottawa, Canada; 4 Centre for Reviews and Dissemination (CRD), Centre for Reviews and Dissemination, University of York, Heslington, York YO10 5DD; 5 West Los Angeles VA Medical Center, 10833 Wilshire Boulevard, 111G, Los Angeles, CA 90073, USA

## 

About a year ago we launched *Systematic Reviews*. During this first year we have been delighted by the journal’s growth and rate of development. At the time of writing we have published 84 articles. Of these, 42 were protocols, 17 were completed systematic reviews, 9 were methods papers, and 16 were other types of articles including two overviews. Twenty articles have been highly accessed, meaning that within a short period of time of being published they were frequently accessed, ranging from 2,000 to 9,000 times. Topics cover a wide spectrum of health issues, such as male participation in prevention programs of mother to child transmission of HIV; psychosocial interventions to reduce alcohol consumption; the use of intravascular ultrasound during bare-metal coronary stent insertion; to the effectiveness of certain antibiotics for preventing traveler’s diarrhea. We have also published a number of articles about evidence synthesis methods: a systematic review of guidelines for clinical trial protocol content, sample size and power consideration in network meta-analysis, and the evolution of approaches to rapid reviews. Corresponding authors have come from many parts of the globe, including Australia, Cameroon, Finland, Germany, and the United Kingdom.

Our launch issue included a series of nine articles about the importance of registering systematic reviews and the development of PROSPERO, an international prospective register of systematic reviews, and we are pleased to stream titles of latest protocols registered in PROSPERO on the journal home page. We are now indexed on Medline, PubMed, and Scopus and will apply to Thomson Reuters ISI for a journal impact factor (JIF) in the near future. Other relevant metrics, such as the number of accesses and citations, as well as an Altmetric score, can be found when accessing any journal article.

Based on data from the journal’s website the journal is gaining momentum and attracting interest from readers. When we launched we had about 3,000 visits per month which has grown more than fivefold to more than 17,000 visits per month. We are very gratified with our early progress and thank all of our authors who took a risk and submitted their research to the journal.

Looking ahead, we see a number of interesting developments happening within the systematic review community that we are interested in featuring in the Journal. Based on the number and trajectory of published network meta-analyses (NMA) and associated methods papers, this type article has gained considerable interest within the systematic review and decision-making communities. *Research Synthesis Methods* recently published a special series on the more technical aspects of NMA [1], and we anticipate a growing number of published networks. The developing field of rapid reviews is of interest to readers, with articles in this area being among the most highly accessed at the journal. For example, the Khangura *et al*. paper [2] on their development of rapid reviews has been downloaded almost 9,000 times since it was published in February 2012. We would like to publish more actual rapid reviews and articles about the different methods of conducting them. Systematic review updates is another area that is gaining attention, both how to do updates and the updates themselves. To date, the journal has published two systematic review updates and is interested in publishing more. Lastly, almost all of the papers we received are reported using the IMRAD format. While this style is likely convenient for authors and to academic readers, we encourage authors to push the envelope. We would like them to experiment with different formats. This is particularly true for systematic review updates. Authors might want to report the results of the original review, their methods, briefly, and results, and what the update provides above and beyond the results from the original systematic review.

We are delighted to be part of the Cochrane Collaboration’s 20th year anniversary. Later this year we will publish a series of articles celebrating the collaboration’s contributions to the science of systematic reviews over the last 20 years. Recently the journal made a call for papers in two areas - the latest developments in comparative effectiveness treatments for obesity and in treatments for dementia; and new developments in the conduct and reporting of diagnostic and prognostic systematic reviews. We hope to publish a series of papers on each topic later this year.

We hope authors will consider the journal to disseminate their research. We remain committed to innovation and to open access, including helping to ensure the widest possible readership for all articles published in the journal.

## Competing interests

Other than being co-editors in chief we have not other competing interests.

## Authors’ contributions

DM drafted the initial paper and PS and LS provided critical revisions. All authors read and approved the final manuscript.
